# The potential risks and impact of the start of the 2015–2016 influenza season in the WHO European Region: a rapid risk assessment

**DOI:** 10.1111/irv.12381

**Published:** 2016-05-14

**Authors:** Raïssa Tjon‐Kon‐Fat, Tamara Meerhoff, Sergejs Nikisins, João Pires, Dmitriy Pereyaslov, Diane Gross, Caroline Brown, WHO European Region Influenza Network, A. Drishti, I. Hasibra, M. Kota, A. Simaku, S. Sarkisian, L. Torosyan, G. El Belazi, C. Hain, P. Lachner, R. Muchl, T. Popow‐Kraupp, M. Redlberger‐Fritz, R. Strauss, N. Abdullayeva, O. Salimov, N. Gribkova, V. Shimanovich, N. Bossuyt, A. Hombrouck, S. Moreels, I. Thomas, . an Casteren, D. Bastinac, A. Dedejic Ljubovic, D. Kojic, M. Kovacevic Suljkanovic, M. Kuzmanovic, N. Vukmir Rodic, T. Georgieva, M. Kojouharova, N. Korsun, V. Drazenovic, M. Erceg, S. Kurecic‐Filipovic, A. Simunovic, V.V. Visekruna, D. Bagatzouni, A. Elia, M. Koliou, M. Havlickova, H. Jirincova, J. Kyncl, K. Bragstad, T. Kolsen Fischer, K.L. Krause, A. Mazick, R. Trebbien, I. Dontsenko, L. Dotsenko, L. Pokras, O. Sadikova, N. Ikonen, O. Lyytikainen, S. Murtopuro, P. Ruutu, S. Behillil, E. Belchior, T. Blanchon, I. Bonmarin, L. Bruno, J.M. Cohen, V. Enouf, B.D. Levy, A. Mosnier, C. Turbelin, M. Valette, . an der Werf, G. Chakhunashvili, A. Machablishvili, K. Zakhashvili, G. Andreas, S. Buda, T. Eckmanns, G. Krause, G. Poggensee, B. Schweiger, A. Kossivakis, N. Malisiovas, A. Mentis, G. Spala, A. Csohan, I. Jankovics, K. Kaszas, Z. Molnar, M. Rozsa, T. Gudnason, A. Löve, G. Sigmundsdottir, S. Coughlan, L. Domegan, M. Duffy, D. Igoe, J. O'Donnell, D. O'Flanagan, A. Waters, Z. Kaufman, M. Mandelboim, A. Bella, I. Donatelli, M.G. Pompa, C. Rizzo, D. Amandosova, A. Kuatbaeva, G. Nusupbaeva, M. Smagulova, M. Smagul, M. Sultanova, D. Otorbaeva, G. Saparova, R. Butirina, R. Nikiforova, J. Storozenko, N. Zamjatina, A. Griskevicius, V. Lipnickiene, S. Muralyte, J. Mossong, M. Opp, C. Barbara, Z. Graziella, M.J. Maistre, T. Melillo, B. Rakocevic, Z. Vratnica, I. Hooiveld, M. de Lange, F. Dijkstra, G. Donker, A. Meijer, G. Rimmelzwaan, A. Teirlinck, W. van der Hoek, S. Dudman, S.H. Hauge, O. Hungnes, A. Kilander, R. Tonnessen, K. Bednarska, L. Brydak, A. Wozniak‐Kosek, A. Zielinski, R. Guiomar, B. Nunes, V. Eder, C. Spinu, V. Alexandrescu, E. Lupulescu, F. Popovici, E. Burtseva, A. Komissarov, E. Smorodintseva, A. Sominina, D. Dimitrijevic, S. Filipovic, E. Staronova, N. Berginc, K. Prosenc, M. Socan, V. Ucakar, M. Grgic Vitek, I. Casas, R. Ortiz de Lejarazu, A. Larrauri, F. Pozo, T. Vega, M. Ali, M. Brytting, H. Dahl, H. Englund, A. Tegnell, A. Wallensten, A. Wiman, R. Born, S. Cordey, M. Kamolov, G. Bosevska, Z. Karadzovski, G. Kuzmanovska, V. Mikik, G. Korukluoglu, S. Topal, A. Ashyrova, G. Ovliyakulova, I. Demchyshyna, T. Dykhanovska, A. Mironenko, O. Blatchford, W. Carman, P. Coyle, R. Gunson, C. Kearns, A. MacLean, J. Mcmenamin, C. Moore, C. Nugent, R. Pebody, N. Phin, A. Reynolds, B. Smyth, J. Watson, M. Zambon, S. Dzemileva, R. Rakhimov

**Affiliations:** ^1^ Division of Communicable Diseases and Health SecurityWHO Regional Office for EuropeCopenhagenDenmark; ^2^ Department of Primary and Community CareRadboud University Medical CenterNijmegenthe Netherlands

**Keywords:** 2015–2016 Influenza season, influenza A(H1N1)pdm09 virus, seasonal influenza, WHO European Region

## Abstract

**Background:**

Countries in the World Health Organization (WHO) European Region are reporting more severe influenza activity in the 2015–2016 season compared to previous seasons.

**Objectives:**

To conduct a rapid risk assessment to provide interim information on the severity of the current influenza season.

**Methods:**

Using the WHO manual for rapid risk assessment of acute public health events and surveillance data available from Flu News Europe, an assessment of the current influenza season from 28 September 2015 (week 40/2015) up to 31 January 2016 (week 04/2016) was made compared with the four previous seasons.

**Results:**

The current influenza season started around week 51/2015 with higher influenza activity reported in Eastern Europe compared to Western Europe. There is a strong predominance of influenza A(H1N1)pdm09 compared to previous seasons, but the virus is antigenically similar to the strain included in the seasonal influenza vaccine. Compared to the 2014/2015 season, there was a rapid increase in the number of severe cases in Eastern European countries with the majority of such cases occurring among adults aged <65 years.

**Conclusions:**

The current influenza season is characterized by an early start in Eastern European countries, with indications of a more severe season. Currently circulating influenza A(H1N1)pdm09 viruses are antigenically similar to those included in the seasonal influenza vaccine, and the vaccine is expected to be effective. Authorities should provide information to the public and health providers about the current influenza season, recommendations for the treatment of severe disease and effective public health measures to prevent influenza transmission.

## Introduction

Each year, seasonal influenza is estimated to affect 5–10% of the world's population resulting in between 250 000 and 500 000 deaths, as well as causing significant costs to health services. In the 2014–2015 influenza season, there was an unusually high excess mortality in the 15 European countries participating in the European monitoring of excess mortality for public health action (EuroMOMO).^1^ An estimated 217 000 premature deaths occurred among the elderly; many of which are likely to be due to influenza.

Countries conduct influenza surveillance to determine when and where influenza activity is occurring, detect changes in the antigenic and genetic characteristics of seasonal influenza viruses, describe the clinical patterns of influenza and risk factors for severe disease, assess the relative severity of the season and detect unusual events due to influenza.^2^ In the World Health Organization (WHO) European Region, this surveillance is coordinated by the WHO Regional Office for Europe and the European Centre for Disease Prevention and Control (ECDC) which jointly publish the weekly influenza update ‘Flu News Europe’ (FNE) between weeks 40 and 20 of each year.^3^

As part of overall surveillance core capacities under the International Health Regulations,^4^ Member States are required to have risk assessment capacity at national level for acute public health events. WHO assists countries in performing risk assessments for events at national level and takes the lead in conducting risk assessments for multicountry or regional events. A risk assessment systematically gathers and assesses information to assign a level of risk to an acute public health event.^5^ WHO guidance on how to perform a rapid risk assessment (RRA) entitled ‘Rapid risk assessment of public health events’ recommends assessing the risk, with respect to the hazard, exposure and context, of an acute public health event.^5^ The RRA is performed by addressing the critical risk questions in order to scope the focus of the risk assessment to key priority concerns that need immediate public health measures. Therefore, RRAs are intended to rapidly support and direct public health decision‐making.

The WHO Regional Office for Europe started receiving reports from a number of Eastern European countries of severe disease and deaths associated with influenza A(H1N1)pdm09 in late December 2015, as well as requests for support (Armenia, Georgia and Ukraine). There was high media attention and concern among the public in these countries.

To investigate whether the 2015–2016 influenza season could be a more severe season compared with previous seasons, a RRA was conducted among the 50 of the 53 Member States of the WHO European Region which provide data to FNE.

## Materials and methods

The RRA was conducted using the WHO manual ‘Rapid risk assessment of acute public health events’ according to a list of risk questions (Table 1).^5^ It is based on data available on FNE between 28 September 2015 (week 40/2015) and up to 31 January 2016 (week 04/2016) as well as historical data from 4 previous influenza seasons (i.e. the 2011–2012, 2012–2013, 2013–2014 and 2014–2015 influenza seasons). It includes information from the WHO risk assessment – seasonal influenza A(H1N1)pdm09 published on 8 February 2016 – and the ECDC risk assessment – seasonal influenza 2015–2016 in the EU/EEA countries published on 8 February 2016.^6^, ^7^ The WHO recommendations arising from this RRA have already been published to support timely and appropriate control measures in Member States of the WHO European Region.

**Table 1:** Overview risk assessment

Component (definition)	Key output	Risk questions
Hazard (identification of a potential hazard that causes the event with adverse effects)	Viral factors	Is there a higher proportion of influenza A(H1N1)pdm09 versus influenza A(H3N2) and influenza B compared with previous seasons?
		Is there evidence that currently circulating influenza A(H1N1)pdm09 viruses have changed antigenically compared with the vaccine virus, or acquired mutations that would result in increased virulence or severity of disease?
		Is there evidence of reduced susceptibility to the neuraminidase inhibitors oseltamivir and zanamivir of currently circulating A(H1N1)pdm09 viruses?
	Clinical factors	Is a particular virus more frequently associated with severe infection?
Exposure (evaluation of the exposure of individuals to potential hazards)	Epidemiology of infection	Is there evidence of a more severe season?
		Is there a difference in the age groups that are severely affected this season?
		Is this season associated with a higher frequency of severe outcomes?
	Susceptibility	Is there a difference in age groups that are severely affected this season? Is there a difference in age groups with/without comorbidities that are severely affected this season?
	Population immunity	In countries experiencing a severe season, are there more persons affected, or affected severely, compared with other countries due to the fact that there are still people that have not been infected with the A(H1N1)pdm09 virus?
	Vaccine effectiveness	Is there evidence of reduced vaccine effectiveness?
	Transmission	Is the start of the season earlier than usual/unusually earlier?
		Are there unusually high levels of influenza activity in the community?
Context (evaluation of the environment of the event)	Socio‐economic	Are there socio‐economic factors that could result in an increase in severe disease this season? Are vulnerable or displaced persons affected?
	Programmatic	Are there more severe cases in some countries due to weak health systems or political or other crisis?
		Could reports of severity in some countries be (partly) explained by changes in surveillance practices due to media attention/concerns of the public?

Based on the WHO manual ‘Rapid risk assessment of acute public health events’.^5^

The WHO European Region consists of 53 Member States, of which 50 have surveillance systems for seasonal influenza. National influenza focal points report data on influenza to FNE through the ECDC platform The European Surveillance System (TESSy) on a weekly basis during the influenza surveillance season (week 40 to week 20 of each year). Data sources vary by country, but include sentinel and non‐sentinel surveillance systems for influenza‐like illness (ILI), acute respiratory infections (ARI), severe acute respiratory infections (SARI) and/or influenza‐confirmed hospitalized cases.^3^

Epidemiological and virological influenza data were included in the current analysis.

**Figure 1:**
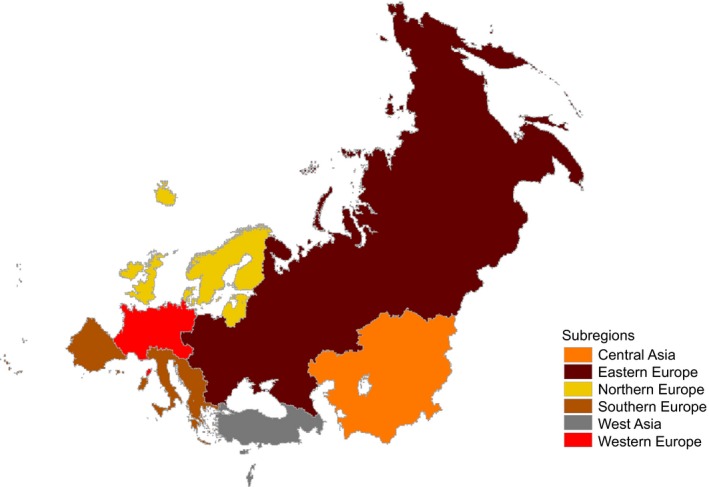
Subregions of the WHO European Region, according to the United Nations geographic composition. *WHO European Region Member States that participate in influenza surveillance grouped into subregions according to United Nations geographic composition (http://unstats.un.org/UNSD/METHODS/M49/M49REGIN.HTM): Western Europe (Austria, Belgium, France, Germany, Luxembourg, Monaco, the Netherlands, Switzerland); Northern Europe (Denmark, Estonia, Finland, Iceland, Ireland, Latvia, Lithuania, Norway, Sweden, United Kingdom); Southern Europe (Albania, Bosnia and Herzegovina, Croatia, Greece, Italy, Malta, Montenegro, Portugal, Serbia, Slovenia, Spain, The former Yugoslav Republic of Macedonia); Western Asia (Armenia, Azerbaijan, Cyprus, Georgia, Israel, Turkey); Eastern Europe (Belarus, Bulgaria, Czech Republic, Hungary, Poland, Republic of Moldova, Romania, Russian Federation, Slovakia, Ukraine); Central Asia (Kazakhstan, Kyrgyzstan, Tajikistan, Turkmenistan, Uzbekistan).

Where appropriate, we grouped countries into Southern, Northern, Eastern and Western Europe, as well as Central and Western Asia subregions, according to United Nations geographic classification (Figure 1).^8^

### Hazard assessment

Data available from FNE were assessed to answer risk questions related to viral factors and clinical factors associated with the specific hazard.

Viral factors included potential antigenic drift compared to the vaccine strains; acquired genetic mutations that could be associated with increased virulence, including evidence of antiviral resistance, based on data available for 20 countries, representing all subregions except Western and Central Asia.

The proportion of A(H1N1)pdm09 viruses among total sentinel ILI and ARI detections and non‐sentinel detections were analysed for the WHO European Region and for the six subregions from week 40/2015 to week 04/2016 of this season. The proportion of A(H1N1)pdm09 viruses among total sentinel ILI and ARI detections from week 40/2015 to week 04/2016 of this season was compared to the same time period from the last four seasons.

### Exposure assessment

In order to assess the epidemiology of the infection, we evaluated the severity of the current season compared with previous seasons for all countries for which it was considered that the season had started. As not all countries had a threshold/baseline for influenza activity, we were unable to use this to assess the start of the season and used instead the criterion of at least five consecutive weeks of increasing influenza activity according to the influenza‐like illness (ILI) or acute respiratory infections (ARI) consultation rates. All countries reporting severe acute respiratory infections (SARI) from sentinel hospitals or laboratory‐confirmed influenza in hospitalized patients were assessed. In total, data from 26 countries were used for this study (Table 2).

**Table 2:** List of countries used in the analyses performed for this study (for influenza‐like illness (ILI), acute respiratory infections (ARI), severe acute respiratory infections (SARI) and/or laboratory‐confirmed hospitalized cases)

Name of country	Influenza‐like illness (ILI)	Acute respiratory infections (ARI)	Severe acute respiratory infections (SARI)	Laboratory‐confirmed hospitalized cases
Albania		✓	✓	
Armenia	✓	✓	✓	
Azerbaijan	✓		✓	
Belarus	✓	✓	✓	
Estonia	✓	✓		
Finland	✓	✓		✓
France	✓			✓
Georgia	✓		✓	
Greece	✓			
Ireland	✓			✓
Israel	✓			
Italy	✓			
Kazakhstan	✓	✓	✓	
Kyrgyzstan	✓	✓	✓	
Portugal	✓			
Republic of Moldova	✓	✓	✓	
Romania	✓	✓		✓
Russian Federation	✓	✓	✓	
Serbia	✓		✓	
Slovakia	✓	✓		✓
Spain	✓			✓
Sweden				✓
Switzerland	✓			
Turkey	✓			
Ukraine	✓	✓	✓	
United Kingdom				
England	✓	✓		✓
Northern Ireland	✓	✓		
Scotland	✓	✓		
Wales	✓			

We evaluated the trend (i.e. increases and decreases) of influenza activity for ILI, ARI and SARI compared with the four previous seasons. Also, we compared SARI rates for this season with the previous season (2014–2015) with respect to the number of cases by age groups, influenza virus type and subtype among cases and the percentage of SARI cases positive for influenza for this and the previous season. The number of cases, the age groups and the subtyped viruses for hospitalized patients with laboratory‐confirmed influenza were also evaluated.

Data for mortality were available through the European monitoring of excess mortality for public health action (EuroMOMO), a mortality monitoring system aimed at detecting and measuring excess all‐cause mortality in real time (weekly) during the influenza season.^9^

Neither data on population immunity to seasonal influenza A(H1N1)pdm09 nor data on vaccine coverage were available publicly for the 2015–2016 season for all countries in the WHO European Region. In order to evaluate the possible impact of seasonal influenza vaccine coverage on the severity of the 2015–2016 season in all 53 Member States, published data on seasonal influenza vaccine coverage from the 2008–2009 and 2009–2010 seasons as well as unpublished data were evaluated 10 (WHO: unpublished data). Interim data on vaccine effectiveness from the Influenza Monitoring Vaccine Effectiveness (I‐MOVE) project for this season (EU/EEA countries only) are also discussed.^11^

### Context assessment

Information on surveillance practices in countries reporting sentinel SARI cases is discussed. However, information on socio‐economic factors that could result in an increase in severe disease this season, or on the extent to which vulnerable or displaced persons may be more severely affected, was not available.

## Results

### Hazard assessment

#### Viral factors

Since week 40/2015 until week 4/2016, 227 572 specimens have been tested from sentinel and non‐sentinel sources in the WHO European Region, of which 14% have tested positive for the influenza virus; 85% of the influenza virus‐positive specimens from sentinel sources and 94% of those from non‐sentinel sources contained influenza A viruses, with a strong predominance of A(H1N1)pdm09 among the viruses subtyped (84% from the sentinel sources and 91% from the non‐sentinel sources).

**Figure 2:**
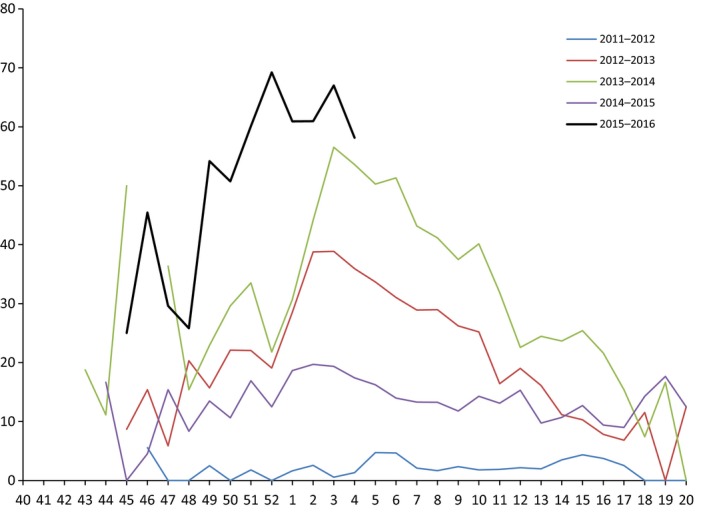
Proportion of A(H1N1)pdm09 among subtyped influenza viruses for sentinel detections. *Visualized data for weeks with >10 detections; week 53/2015 is excluded from the graph.

The proportion of A(H1N1)pdm09 viruses among total subtyped sentinel ILI/ARI specimens exceeded 50% in all weeks between weeks 49/2015 and 04/2016, with the highest proportion (70%) reported in week 52/2015. During four previous seasons, a proportion of A(H1N1)pdm09 of more than 50% was reported in one week only, namely week 03/2014, while for the other three seasons, the proportion of A(H1N1)pdm09 remained below 40% between weeks 49 and 4 (Figure 2).

This season, there are geographic differences in the proportion of influenza A and B virus types and subtypes that were reported (Table 3). All geographic subregions reported predominantly influenza A, and of influenza A subtyped viruses, the A(H1N1)pdm09 were predominant. However, Western Europe reported 60% influenza A and 40% influenza B, while the other subregions reported between 89–100% A. Southern, Eastern, Northern Europe and Central Asia reported between 89–97% A(H1N1)pdm09. Western Asia reported the largest proportion of A(H3N2) (26%) among A subtyped viruses (Table 3).

**Table 3:** Distribution of influenza detections in the WHO European Region: 2015–2016 season (weeks 40/2015 to 04/2016)

Influenza detections^a^	Southern Europe		Western Europe		Eastern Europe		Northern Europe		West Asia		Central Asia		WHO European Region	
Influenza A	3355	91%	1789	60%	8104	98%	10 001	89%	5926	89%	455	100%	29 630	89%
Influenza A subtyped	2308		966		7464		4117		5899		352		21 106	
A (H1N1)pdm09	2069	90%	836	87%	7207	97%	3959	96%	4372	74%	312	89%	18 755	89%
A (H3N2)	239	10%	130	13%	257	3%	158	4%	1527	26%	40	11%	2351	11%
Influenza B	319	9%	1177	40%	135	2%	1232	11%	764	11%	0	0%	3627	11%
B lineage determined	67		232		22		185		0		0		506	
B‐Yamagata lineage	6	9%	14	6%	6	27%	49	26%	0		0		75	15%
B‐Victoria lineage	61	91%	218	94%	16	73%	136	74%	0		0		431	85%
Total	3674		2966		8239		11 233		6690		455		33 257	

a Combined ILI, ARI sentinel and non‐sentinel respiratory specimens positive for influenza.

Across the region, 11% of viruses were influenza B. Although lineage determination was performed on only a subset of influenza B viruses, the Victoria lineage was dominant (85% of the total number of lineage‐determined specimens positive for influenza type B). Of the 3627 influenza B detections reported between week 40/2015 and week 04/2016, the B‐Victoria lineage was assigned to 431 viruses and the B‐Yamagata lineage to 75 (Table 3).

Antigenic characterization data from 447 influenza viruses reported by National Influenza Centres (NICs; data were available from all subregions except Western and Central Asia) to FNE showed that the majority (95%) of these were similar to the viruses recommended for trivalent and quadrivalent vaccines for use in the 2015–2016 influenza season. This included all 381 antigenically characterized A(H1N1)pdm09 viruses.

So far this season, phylogenetic analysis of the haemagglutinin (HA) of A(H1N1)pdm09 viruses demonstrated that these viruses belong to genetic subgroup 6B, in common with most A(H1N1)pdm09 viruses characterized from more than 30 countries worldwide since September 2015.^6^

Of the 466 A(H1N1)pdm09, 55 A(H3N2) and 47 type B viruses tested for neuraminidase inhibitor susceptibility since week 40/2015, only two A(H1N1)pdm09 viruses showed highly reduced inhibition by oseltamivir associated with NA‐H275Y amino acid substitution; the remainder showed no molecular or phenotypic evidence of reduced inhibition by neuraminidase inhibitors.

#### Clinical factors

Of the subtyped influenza A viruses, in countries reporting SARI as well as laboratory‐confirmed influenza in hospitalized or intensive care units, the vast majority were influenza A(H1N1)pdm09. Of the 564 influenza A viruses that have been subtyped, there is a predominance of A(H1N1)pdm09 viruses this season in influenza‐confirmed hospitalized cases (Finland, France, Ireland, Romania, Slovakia, Spain, Sweden and United Kingdom – England) (96%, range 92–100%). For France and Ireland, there was also an influenza B cocirculation in influenza‐confirmed hospitalized cases this season, and the proportion of influenza B was 20% and 38%, respectively.

### Exposure assessment

#### Epidemiology of infection

**Figure 3:**
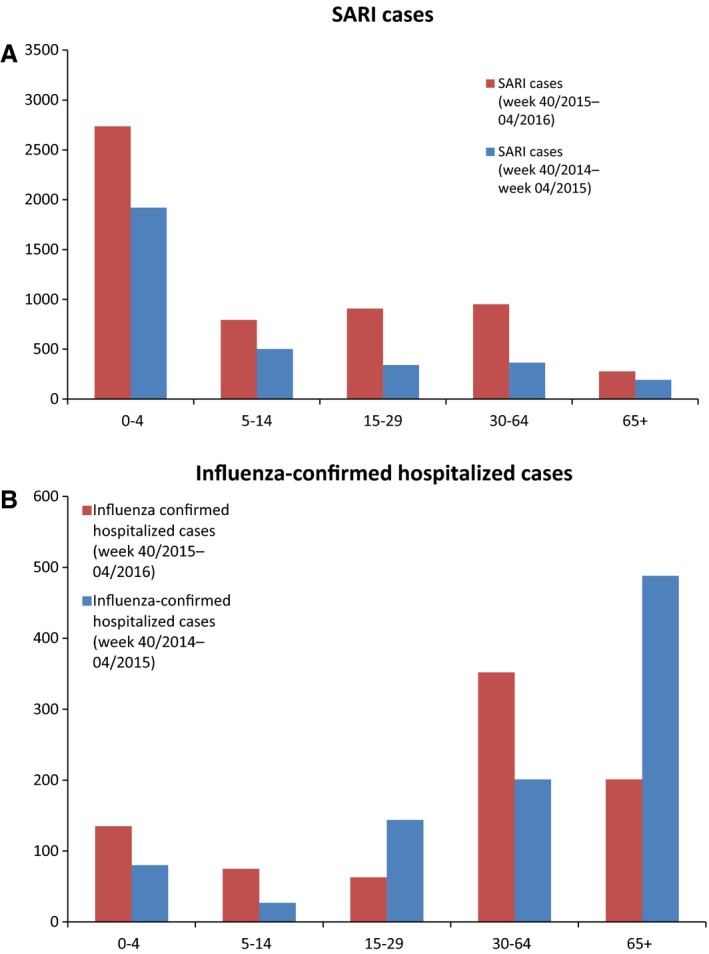
(A) Age groups of SARI cases for the 2014–2015 and 2015–2016 season week 20 to week 4. (B) Age groups of influenza‐confirmed hospitalized cases for the 2014–2015 and 2015–2016 season week 40 to week 4.

Five countries (Armenia, Georgia, Russian Federation, Serbia and Ukraine) reporting data on SARI cases had sufficient data for comparison with the 2014–2015 season. Up until week 4/2016, there was an increase in severe disease (SARI cases) compared to 2014–2015. The number of SARI cases increased in all age groups compared with the previous season, but the proportion of adults younger than 65 rose and shows a slightly different trend compared to the 2014–2015 season (Figure 3A).

Only five of the eight countries that report on influenza‐confirmed hospitalized cases had data on age groups for this and the previous season (Spain, France, Ireland, Romania and Sweden). For this season, the majority of cases are in those aged 20–64, while last season this was the 65 and older group (Figure 3B).

However, data on mortality from the 18 EU/EEA countries and regions in Western, Northern and Southern Europe that report to EuroMOMO show that so far this season, there have been no indications of an increase in excess all‐cause mortality.^9^

#### Susceptibility

Seven of eight countries reporting influenza‐confirmed hospitalized cases had data on underlying comorbidities. However, this amounted to only 14% of cases, precluding further analysis.

#### Population immunity and vaccine effectiveness

Currently available data on seasonal influenza vaccine policies and coverage in all 53 Member States show low coverage in the elderly among many countries (WHO: unpublished data).^10^ The vaccination coverage target of 75% in the elderly population by 2010 was achieved by two countries (the Netherlands and United Kingdom – Northern Ireland and Scotland).^12^ Furthermore, mechanisms to monitor vaccination coverage in target groups other than the elderly are lacking in many countries, impeding accurate assessment of the impact of national influenza vaccination programmes on the 2015–2016 influenza season.

Interim estimates of influenza vaccine effectiveness in Europe published recently by the I‐MOVE Project show an adjusted vaccine effectiveness of 46·3% (95% CI: 4·9–69·7%).^11^

#### Transmission

Using a threshold of ten per cent influenza positive among specimens from sentinel surveillance sources for the WHO European Region, the season started in week 51/2015; 46% of the Member States were reporting low‐intensity influenza activity in week 4/2016, which was similar to the previous season when 50% of countries reported low‐intensity influenza activity for week 4/2015 (Figure S1). When comparing the influenza intensity reported in week 4 for this season with the previous season, there was a variation by subregion. For Southern and Western Europe, there was low‐intensity influenza activity with 93% of countries reporting medium‐ to high‐intensity influenza activity for 2014–2015 and 53% for 2015–2016. More countries reported medium‐ to very high‐intensity influenza activity in Eastern and Northern Europe and Western Asia (32% of countries for 2014–2015 and 65% for 2015–2016). Reports by Central Asian countries showed a trend similar to last season, only reporting low‐intensity influenza activity.

**Figure 4:**
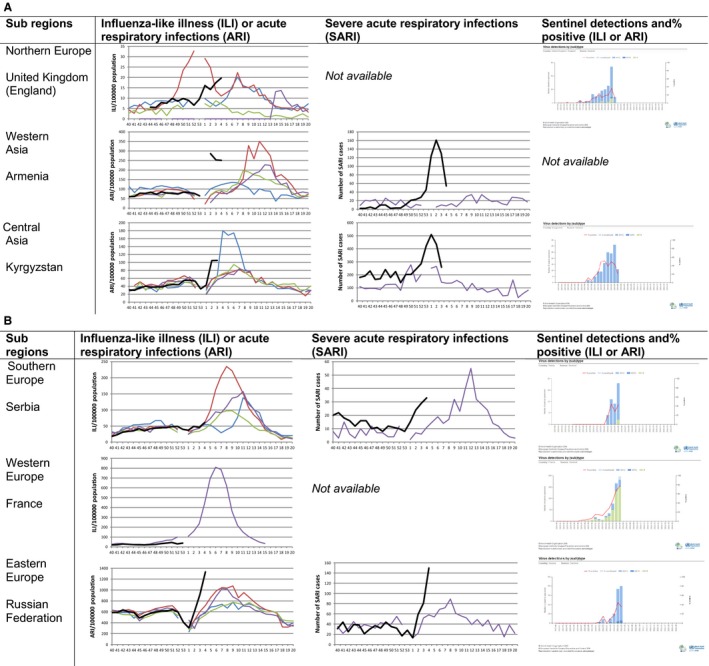
(A and B) Influenza activity for a selection of countries of different subregions (ILI or ARI, SARI and graph of sentinel detections). *For ILI and ARI, the rates are presented per 100 000 population. Black is the 2015–2016 season, purple 2014–2015, green 2014–2014, red 2012–2013 and blue 2011–2012. ** For SARI, the number of SARI cases is presented. Black is the 2015–2016 season, and purple is the 2014–2015 season. *** For the graphs on per cent positive, we have used the graphs available on Flu News Europe that have data until week 5.

When examining the ILI and/or ARI consultation rates for this season, these were similar compared with previous seasons. In five countries reporting SARI (Armenia, Georgia, Russian Federation, Serbia and Ukraine), the increase in SARI cases was steeper and occurred between three to six weeks earlier than the previous season. The trend of influenza activity is illustrated in Figure 4 for a selection of countries from each subregion.

### Context assessment

Regarding surveillance practices, of the five countries from which SARI data were evaluated, three (Armenia, Georgia and Russian Federation) collect respiratory specimens from most or all SARI cases during an influenza season, one (Serbia) adjusts the sampling strategy according to the capacities of the NIC and one (Ukraine) has a specified sampling strategy.^13^ Informal reports from two countries described backlogs in testing due to a much larger number of specimens being collected from SARI cases compared with the previous 2014–2015 season. Information on socio‐economic factors that could result in an increase in severe disease this season, or on the extent to which vulnerable or displaced persons may be more severely affected, was not available.

## Discussion and conclusions

### Main conclusion

In this risk assessment, we evaluated the hazard, exposure and context of seasonal influenza for the start of the 2015–2016 season to assess whether there are indications that this will be a more severe influenza season in comparison with previous season(s). This risk assessment showed that up to week 4/2016, there is a very high predominance of influenza A(H1N1)pdm09, particularly in Eastern Europe, but that these viruses are antigenically similar to the virus included in the 2015–2016 Northern Hemisphere seasonal influenza vaccines and are susceptible to neuraminidase inhibitors. Genetic changes observed in currently circulating A(H1N1)pdm09 viruses are being monitored closely by the WHO GISRS. Furthermore, the season seems to have started earlier in Eastern European countries, which is different to a west–east spread pattern seen in several previous seasons.^14^ Also, a sharper rise in severe cases was seen in these countries compared with the previous season. In the WHO European Region, influenza A(H1N1)pdm09 was associated with severe disease in all age groups. In line with previous findings when A(H1N1)pdm09 is circulating throughout the region, severe cases occurred in greater numbers in adults under 65 years compared with the previous season.

### Strengths and limitations

We performed a rapid but in‐depth risk assessment of the severity of the current influenza season for the WHO European Region. Although the data originate from a diversity of influenza surveillance systems, and some data gaps and quality issues prevented analysis of some data sets, work carried out in close collaboration with the influenza network in the WHO European Region on surveillance standards and country profiles allowed for improved interpretation of the data over the years.^2^, ^15^

This RRA is based on interim data and reflects the current risk in the WHO European Region. However, because the 2015–2016 influenza season is still ongoing, it is difficult to predict the extent or impact of the remainder of the season. This will need to be evaluated as additional data emerge.

We found the WHO risk assessment methodology useful to rapidly assess the risk of the current influenza season. The risk questions that formed the basis for the risk assessment can be adapted as a season progresses, for future influenza seasons and in an influenza pandemic. Questions that could not be answered for this risk assessment, for example the presence or absence of risk factors in persons with severe disease, highlighted some gaps in surveillance, which will be useful to inform future collaboration on influenza surveillance in the WHO European Region.

### Hazard

The current influenza season has shown a very high proportion of influenza A(H1N1)pdm09 viruses across most of the WHO European Region so far, which was higher than during the same period of the four previous seasons.

The WHO has recommended that trivalent vaccines for use in the 2015–2016 influenza season in the Northern Hemisphere contain an A/California/7/2009 (H1N1)pdm09‐like virus, an A/Switzerland/9715293/2013 (H3N2)‐like virus and a B/Phuket/3073/2013‐like virus.^16^ So far, there is no evidence that currently circulating influenza viruses have changed antigenically compared with the vaccine virus. However, of those influenza B viruses for which the lineage has been determined, the predominant lineage is Victoria, which is recommended for inclusion in quadrivalent seasonal influenza vaccines, but not in trivalent vaccines.

Furthermore, we find no evidence, so far, of reduced susceptibility of the influenza viruses from the European Region to the neuraminidase inhibitors oseltamivir and zanamivir.^3^

### Exposure

The current influenza season in the WHO European Region started around week 51/2015, which is similar to previous seasons. Higher influenza activity reported in countries of Eastern and Northern Europe and Western Asia could suggest a different pattern of spread than the west–east spread seen in previous seasons. Of the 5 countries reporting earlier rises in SARI cases compared with the 2014–2015 influenza season, cases peaked first in Armenia in week 01/2016, a country neighbouring the Islamic Republic of Iran which is also experiencing a season predominated by A(H1N1)pdm09 and in which influenza virus detections peaked in week 50/2015. This could support an alternative geographic pattern of spread, possibly from Southern Asia into countries of the WHO European Region this season.^17^

A slightly earlier rise than seen in recent years in severe cases may have caused heightened concern in the affected countries leading to reports of a more severe season. However, as these results are interim and as the current season is still ongoing, it is not possible to say whether this season will be more severe (i.e. increase in the total number of severe cases) compared to the previous season.

This season, influenza A(H1N1)pdm09 was associated with severe disease in all age groups. However, in countries reporting sentinel SARI cases and/or laboratory‐confirmed influenza in hospitals and intensive care units this season, the majority occurred in adults younger than 65 years. This is similar to what has been reported previously for this virus.

Information on comorbidities was only available for a few cases in the region this season, so no further analysis could be performed. However, in the USA, in which influenza A(H1N1)pdm09 is predominating this season, preliminary clinical findings from the Influenza Hospitalization Surveillance Network based on 349 (38·9%) cases show that the majority (90·7%) of hospitalized adults (≥18 years) had at least one reported underlying medical condition; the most commonly reported were cardiovascular disease, metabolic disorders and obesity.^18^

Historically, the uptake of seasonal influenza vaccine varies considerably in countries of the WHO European Region, being generally lower among the elderly in Eastern versus Western European countries. For other risk groups, few countries have systems in place to monitor vaccine uptake.^10^ Low vaccination uptake may increase the risk of persons experiencing complications following influenza infection and contribute to a more severe season in some countries.

Interim estimates of influenza vaccine effectiveness in Europe published recently by the I‐MOVE are of moderate vaccine effectiveness.^11^ However, these are interim results based on a small sample size and should be interpreted with caution. More accurate estimates are expected to be available at the end of the season.

### Context

At least two countries reporting SARI cases experienced a backlog in testing due to a much larger number of specimens being collected compared with the previous 2014–2015 season. Although this did not affect the interpretation of data for this RRA, it would prohibit assessing in real time the proportion of SARI cases that are positive for influenza and whether this was due to a change in adherence by clinicians to the SARI case definition. Such changes in surveillance practice might be fuelled by media reports of a severe season due to a more virulent A(H1N1)pdm09 virus and public concerns.

## Conclusions and recommendations

In conclusion, a possibly earlier start of the season in Eastern European countries, a sharper rise in severe cases and a higher proportion of adults aged <65 years frequently affected led to a more severe start to the season in Eastern European countries. As the season is ongoing, it is difficult to say whether this will be a more severe season compared with the previous seasons for the WHO European Region. So far, there are indications that due to a predominance of influenza A(H1N1)pdm09 seasonal influenza this season, there was a slightly earlier and more severe start of influenza season in Eastern Europe.

A full list of WHO recommendations concerning seasonal influenza is available on the WHO website.^19^ Healthcare personnel should suspect influenza in persons at higher risk of developing severe disease due to influenza (pregnant women, individuals with certain chronic diseases, elderly persons, residents of institutions for older persons and the disabled and children under the age of five years) who present with a clinically compatible illness; sufficient influenza antiviral drugs should be available for treatment. Furthermore, as there is a high predominance of A(H1N1)pdm09, they should have a higher index of suspicion for influenza A(H1N1) when adults younger than 65 present to the healthcare services with severe respiratory disease. Particularly in countries in which the season has not yet started or peaked, it is recommended to provide risk groups with seasonal influenza vaccine. To better inform public health actions during the influenza season, Member States are encouraged to strengthen their existing surveillance systems and existing emergency response capacities.

## Disclaimer

The authors alone are responsible for the views expressed in this article, and they do not necessarily represent the views, decisions or policies of the institutions with which they are affiliated. The boundaries and names shown and the designations used in this publication do not imply the expression of any opinion whatsoever on the part of the World Health Organization concerning the legal status of any country, territory, city or area or of its authorities, or concerning the delimitation of its frontiers or boundaries.

## Conflict of interest

None declared.

## Author's contributions

All authors provided critical discussion of the work and drafting of the manuscript. CB conceptualized the risk assessment and formulated the risk questions. TM and RT analysed the clinical data, and SN and DP analysed the virological data. RT and CB drafted the first draft of the manuscript.

## Supporting information


**Figure S1.** Intensity of influenza activity and dominant virus (sub)type.
