# Supplementary figures and images for: The potential risks and impact of the start of the 2015–2016 influenza season in the WHO European Region: a rapid risk assessment
Source: Influenza Other Respir Viruses. 2016 May 14;10(4):236–46. doi: 10.1111/irv.12381 (PMC4910174; doi:10.1111/irv.12381)

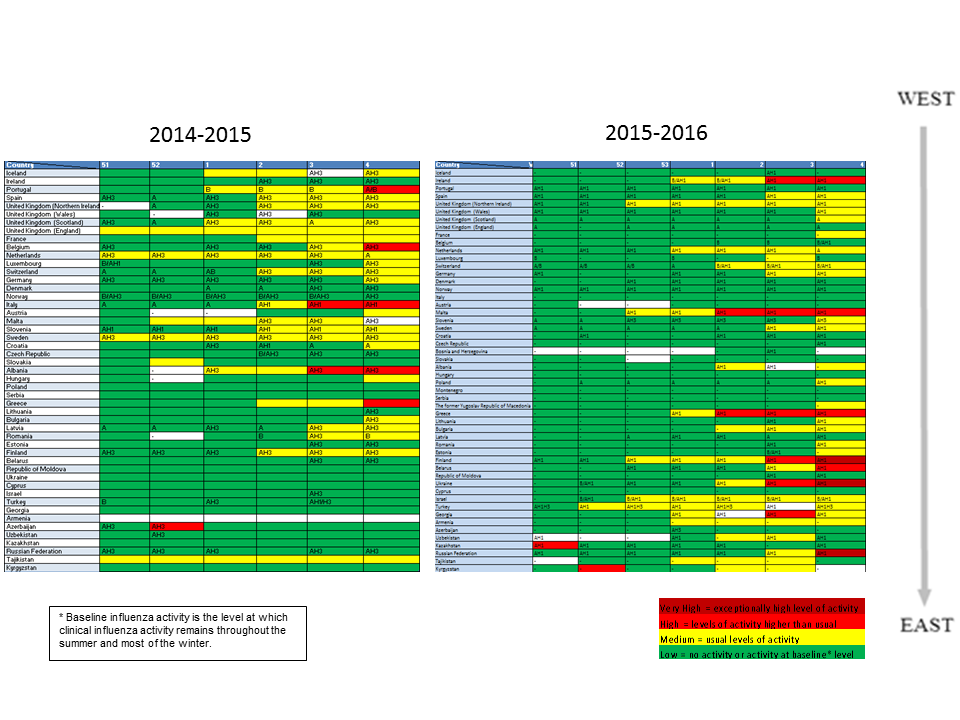

Supplement: Supplementary file 1 — Figure S1. Intensity of influenza activity and dominant virus (sub)type. [file IRV-10-236-s001.tif]
